# Left atrial function after epicardial pulmonary vein isolation in patients with atrial fibrillation

**DOI:** 10.1007/s10840-017-0290-2

**Published:** 2017-11-10

**Authors:** Louise Bagge, Per Blomström, Lena Jidéus, Stefan Lönnerholm, Carina Blomström-Lundqvist

**Affiliations:** 1 Department of Cardiology, Institution of Medical Sciences, Uppsala University, 751 85 Uppsala, Sweden; 2 Department of Cardiothoracic Surgery, Institution of Surgical Sciences, Uppsala University, Uppsala, Sweden

**Keywords:** Atrial function, Atrial fibrillation, Surgical ablation

## Abstract

**Purpose:**

Epicardial pulmonary vein isolation has become an increasingly used therapy for medically resistant atrial fibrillation. The purpose of the present study was therefore to evaluate if epicardial pulmonary vein isolation combined with ganglionated plexi ablation affects the size and mechanical function of the left atrium, and whether the effects are dependent on the extensiveness of the ablation applications.

**Methods:**

A total of 42 patients underwent an echocardiographic examination prior to and 6 months after a minimal invasive epicardial pulmonary vein isolation procedure for the assessment of the effects on left atrial size and function. In 27 patients, who had sinus rhythm both at baseline and follow-up, was a comparison of atrial size and function possible at these time intervals. Fractional area changes were obtained from the left atrial end-systolic and end-diastolic areas in the apical four-chamber view. Pulsed-Doppler was used to assess the transmitral flow velocities to evaluate mechanical function.

**Results:**

Left atrial size and function at 6-month follow-up had not changed significantly from those at baseline as indicated by left atrial maximal area (17.1 ± 4.6 vs. 18.7 ± 5.3, *p* = 0.118), minimal area (12.5 ± 3.8 vs. 13.4 ± 4.7, *p* = 0.248), fractional area change (27.4 ± 8.2 vs. 28.7 ± 10.6, *p* = 0.670), and E/A ratio (1.49 ± 0.47 vs. 1.54 ± 0.67, *p* = 0.855).

**Conclusions:**

Radiofrequency ablation for epicardial pulmonary vein isolation combined with ganglionated plexi ablation has no major effects on atrial function or size. A preserved atrial function for those maintaining sinus rhythm may have important implications for thromboembolic risk after surgery, but warrants confirmation in larger trials.

## Introduction

When choosing the most optimal interventional treatment for patients with atrial fibrillation (AF), not only the success rate in eliminating AF, but also the potential risks for complications, such as adverse effects on adjacent structures, should be considered. It is already confirmed that the Cox maze III procedure results in a sustained decrease in atrial contractility [1, 2] but the effect of transvenous catheter-based pulmonary vein (PV) isolation on left atrial (LA) function is unclear and study results are conflicting [3–8]. Preserved atrial function may have several important implications, an essential contribution to the ventricular myocardial performance and a lower rate of AF recurrences and thromboembolic events.

Epicardial PV isolation off-pump combined with ganglionated plexi (GP) ablation has been used for the treatment of AF [9–14]. Although several studies have reported high success rates ranging between 74 and 90% in eliminating AF [10–14], the information about the performance of the left atrium after this type of surgery is still limited. The purpose of the present study was therefore to assess the effects on size and mechanical function of the left atrium of combined epicardial PV isolation and GP ablation, and its relation to the extensiveness of the ablation applications.

## Methods

### Patients and investigations

The patients selected for the surgical procedure were all symptomatic patients with paroxysmal, persistent or longstanding persistent AF who had either failed catheter ablation or had chosen this option as first alternative. All patients had failed at least one class I (86%) or class III (93%) antiarrhythmic agent. A total of 42 patients who underwent minimal invasive epicardial PV isolation combined with GP ablation (mEPVI-GPabl) [9] during November 2005 and March 2008 were evaluated with echocardiography prior to and 6 months after surgery. In three patients, the baseline study was limited to a transesophageal echocardiography, precluding a comparison of LA area and E/A ratios at 6 months, thus leaving 39 patients for the echocardiographic analysis. In nine patients, the presence of AF at baseline and in three others at 6 months precluded comparisons during the same rhythm before and after surgery, leaving 27 patients for such an analysis. The patient demographics are listed in Table 1.

**Table 1:** Patient demographics

Characteristics	*n* = 27
Age, yrs., mean ± SD	57.7 ± 8.9
Sex, male	17 (63)
Duration of AF, yrs., mean ± SD	8.1 ± 4.9
Paroxysmal AF	22 (81)
Persistent AF	5 (19)
Permanent AF	0 (0)
Lone AF	13 (48)
Hypertension	9 (33)
Cerebral stroke/TIA	8 (30)
Diabetes mellitus	0 (0)
Coronary artery disease	1 (4)
Valvular heart disease	3 (11)
Heart failure or cardiomyopathy	2 (7)
Asthma	4 (15)
Sleep apnea syndrome	1 (4)
LVEF < 50%	2 (7)
LAd, cm, mean ± SD	4.4 ± 0.72

Figures denote numbers and percentages in brackets. *AF*, atrial fibrillation; *yrs*., years; *TIA*, transient ischemic attack; *LVEF*, left ventricular ejection fraction; *LAd*, left atrial diameter

The outcome and clinical evaluation at baseline and follow-up has previously been described in detail [9]. Twelve (44%) of the patients had previously failed a transvenous catheter-based PV isolation, and five patients (19%) had previously undergone a tricuspid isthmus-dependent atrial flutter ablation. Four patients (15%) had a permanent pacemaker preoperatively, although none of the patients were paced in the ventricles. Three of these patients had sick-sinus syndrome and an AAI pacemaker, which may have resulted in delayed LA activation but unlikely with any impact on LA function. The last patient had a DDD pacemaker implanted for bradycardia protection, but it was not in use. Antiarrhythmic drugs were withdrawn in patients who remained in sinus rhythm at 3 months after surgery.

### Procedural technique

The minimal invasive off-pump procedure has been described previously [9] and included epicardial PV isolation, LA GP ablation, division of the ligament of Marshall if identified (96%), and left atrial appendectomy. The bipolar radiofrequency (RF) ablation clamp used for PV isolation was applied well proximal to the confluence of the veins at the antrum, and RF energy was applied according to an algorithm that automatically stopped the ablation when the impedance change indicated that a transmural lesion was achieved. In case conduction block was not achieved after the first three consecutive applications, 1–2 additional RF applications were given until PV conduction block occurred.

After the PV isolation, GP activity was still present in 14 of the patients (52%) defined as bradycardia response to high-frequency electrical stimulation. The activity was abolished by electrocautery or RF ablation until no vagal response could be provoked. In nine patients (33%), RF ablation could not abolish all vagal response. The number of applications for vagal denervation ranged from 1 to 14 applications (median 1). Isolation of all PVs was completed in all but 3 of the 27 patients and required 3 to 4 applications (median 3) on the right side and 2 to 4 applications (median 3) on the left side. PV isolation could not be achieved in one patient despite seven applications. In two cases, right PV isolation could not be performed because of bleeding. The ablation procedure was considered extensive if there were either six or more RF applications at the PV and/or more than ten GP ablations.

The LA appendage was excised using a stapler, if judged feasible and safe according to the surgeon, in 22 of the 27 patients (81%). It was not excised if there was a wide base or thin wall. Complications have been reported elsewhere [9].

### Echocardiographic examination

The echocardiographic examinations with measurements of the left ventricular ejection fraction (LVEF), LA diameter and valve function were made by an experienced technician supervised by a cardiologist. The LVEF was measured in the four-chamber view by Simpson’s method. The LA anterior posterior diameter was measured by M-mode in the parasternal long-axis view, and valve function was evaluated by Doppler flows. Maximal LA cavity areas were obtained by planimetry in the apical four-chamber view at the end of ventricular systole, defined as the last frame before mitral valve opening. Minimal LA cavity areas were obtained at end diastole at the time of the R-wave on the electrocardiogram (ECG). The mean values were calculated from two to three consecutive beats. The atrial fractional area change (FAC) (maximum area − minimum area ∕ maximum area × 100) of the LA was then calculated. Pulsed-Doppler echocardiography was used to assess the transmitral flow velocities from an apical four-chamber view with a sample volume for the tip of the mitral leaflets during diastole. Peak velocities of the early filling deceleration time of the E-wave were measured and averaged over two beats and the E/A ratios were calculated.

### Statistical analysis

All values are expressed as mean ± SD unless otherwise stated. Continuous variables for each patient at different time periods were compared with the use of Wilcoxon matched-paired test. To analyze differences in LA function between patients with enlarged LA and those with normal-sized LA, the ANOVA test was used. The ANOVA test was also used to analyze differences in LA function between patients with extensive PV and/or GP ablations (either six or more PV applications and/or more than ten GP ablations) compared with patients with less extensive PV and GP ablations. Statistica was used for the analyses.

## Results

The outcome and clinical evaluation at baseline and follow-up has been described previously in detail [9], but in brief, 76% patients had no symptomatic AF recurrences or AF episodes on 24**-**h Holter recordings after surgery at 12 months follow-up. One patient with longstanding persistent AF who suffered from a stroke 2 days after surgery had warfarin discontinued 4 days before surgery and did inadvertently not receive low-molecular-weight heparin until the day before operation.

### Atrial dimensions

At baseline, the median LA diameter was 4.3 cm (range 2.6–5.9 cm), and 5 out of 27 patients had enlarged LA, as defined by an equation for predicting normal echocardiographic measurements from body weight and age by Henry et al. [15]. At 6 months follow-up after surgery, the LA diameter decreased or normalized in 3 of these 5 patients with enlarged baseline LA and became enlarged in 2 of the 22 other patients, with normal LA diameter prior surgery.

The LA max and min areas recorded by echocardiography at 6 months follow-up did not differ significantly from those obtained at baseline (Table 2).

**Table 2:** Left atrial areas, fractional area change, and transmitral pulsed-Doppler velocities in patients with sinus rhythm at baseline and 6 months after surgery and at 6 months only in patients with atrial fibrillation at baseline

	SR at baseline and FU (*n* = 27)			AF at baseline (*n* = 9)
Left atrium	Baseline	6 mo	*p* value	6 mo (SR)
Max area cm^2a^	18.7 ± 5.3	17.1 ± 4.6	0.110	21.4 ± 3.7
Min area cm^2a^	13.4 ± 4.7	12.5 ± 3.8	0.248	16.6 ± 4.2
FAC^a^	28.7 ± 10.6	27.4 ± 8.2	0.670	23.5 ± 9.0
E-wave^b^	54.3 ± 13.5	53.5 ± 13.7	0.869	48.8 ± 12.6
A-wave^a^	39.4 ± 11.5	37.3 ± 10.1	0.584	35.1 ± 11.4
E/A ratio^a^	1.54 ± 0.67	1.49 ± 0.47	0.855	1.66 ± 1.19

*SR*, sinus rhythm; *FU*, follow-up; *AF*, atrial fibrillation; *FAC*, fractional area change; *mo*, months

^a^ *n* = 23

^b^ *n* = 26

The LA max and min area recorded during sinus rhythm at 6 months follow-up in patients who had AF at baseline was larger than those in patients with sinus rhythm at baseline, but the difference did not reach statistical significance (Table 2).

A significant decrease in LA maximal area was, however, observed after surgery for patients with enlarged LA at baseline as opposed to patients with normal-sized LA (Table 3). The LA maximal and minimal area change in patients with extensive PV and/or GP ablation lesions (either six or more PV applications and/or more than ten GP ablations) did not differ from those in patients with less extensive PV and/or GP ablations (Table 4).

**Table 3:** Effects of surgery on left atrial areas, fractional area change, and E/A ratio from baseline to 6 months follow-up in patients with enlarged left atrium compared with patients with normal-sized atrium at baseline

	Normal preop LAd (*n* = 18)			Enlarged preop LAd (*n* = 5)			*p* value
	Baseline	6 mo	∆	Baseline	6 mo	∆	
Max area, cm^2^	17.0 ± 2.8	16.4 ± 3.9	− 0.55 ± 3.0	25.0 ± 7.8	19.8 ± 6.4	− 5.15 ± 6.4	.029
Min area, cm^2^	12.1 ± 3.3	11.8 ± 3.2	− 0.25 ± 3.7	18.4 ± 6.1	14.8 ± 5.1	− 3.6 ± 3.5	.082
FAC	29.4 ± 11.6	28.1 ± 8.1	− 1.31 ± 12.9	26.4 ± 6.2	24.7 ± 8.7	− 1.69 ± 11.5	.952
E/A ratio	1.49 ± 0.66^a^	1.44 ± 0.46^a^	− 0.04 ± 0.78	1.90 ± 0.74^b^	1.82 ± 0.50^b^	− 0.08 ± 0.76	.931

The *p* value is tested whether the differences from baseline to 6 months differ significantly between those with normal preop LAd versus those with enlarged LAd

*Preop*, preoperatively; *LAd*, left atrial diameter; *FAC*, fractional area change; *mo*, months

^a^ *n* = 20

^b^ *n* = 3

**Table 4:** Left atrial areas and fractional area changes, at baseline and 6 months follow-up, in patients with non-extensive PV and GP ablation as compared with those with extensive PV and GP ablation after surgery

	Non-extensive ablation (*n* = 12)			Extensive ablation (*n* = 7)			*p* value
	Baseline	6 mo	∆	Baseline	6 mo	∆	
Max area, cm^2^	18.8 ± 3.1	18.9 ± 3.6	0.07 ± 3.5	19.2 ± 8.7	16.4 ± 5.7	− 2.79 ± 3.4	.097
Min area, cm^2^	13.3 ± 4.0	13.7 ± 3.4	0.34 ± 4.2	14.8 ± 6.5	11.9 ± 4.8	− 2.89 ± 1.9	.073
FAC	30.5 ± 11.5	28.3 ± 8.6	− 2.14 ± 13.9	22.7 ± 6.5	27.8 ± 8.2	5.09 ± 7.9	.227

The *p* value is tested whether the differences from baseline to 6 months differ significantly between those with normal non-extensive ablation versus those with extensive ablation

*PV*, pulmonary vein; *GP*, ganglionated plexi; *FAC*, Fractional area change; *mo*, months

### Atrial mechanical function

Overall, the measurements of FAC, E-wave and A-wave velocities, and E/A ratio recorded at 6-month follow-up were not significantly different from those at baseline (Table 2). The FAC and E/A ratio changes in patients with enlarged left atrium at baseline and 6 months did not differ from those in patients with normal-sized left atrium at baseline and 6 months (Table 3). The FAC change after surgery did not differ in patients with extensive PV and/or GP ablations as compared with those in patients with less extensive PV and GP ablation (Table 4).

The E/A ratio had a restrictive pattern in 10 of the 27 patients at baseline, but normalized in six of these patients at 6 months follow-up. Two (20%) of these 10 patients with restrictive LA pattern and three (18%) of the 17 patients with normal E/A ratio had persistent AF. At 6 months follow-up, 9 of 24 patients had a restrictive pattern in the E/A ratio, but only one of them had been subject to extensive PV and/or GP ablations.

None out of the two patients with decreased LVEF at baseline improved their LVEF at 6 months follow-up. One patient developed a slightly impaired LVEF after 6 months despite being in sinus rhythm.

## Discussion

In the past decade, several new surgical AF ablation procedures have been developed, all of which may result in a reduced atrial mechanical function related to extensive scarring of the left atrium, but the effect on LA function has not been systematically studied. Apart from the fact that atrial contractility is an essential contribution to ventricular myocardial performance, that may have important implications for the well-being of the patient, a reduced contractility may also promote thromboembolic events even if AF is successfully eliminated. It is therefore important to assess the LA function when introducing new non-pharmacological procedures for patients with AF.

To the best of our knowledge, the present study is the first to investigate the LA function after epicardial PV isolation combined with GP ablation as a stand-alone procedure.

After the more complex and extensive Cox maze III procedure, a sustained reduction of the atrial mechanical function, by echocardiographic measures of atrial area fractional change and E/A ratios, was reported for patients with paroxysmal lone AF [2, 1]. The clinical impact of studies showing severely compromised LA function may warrant a more restrictive attitude to refer patients with paroxysmal AF for such surgical procedures. Catheter-based RF ablation of AF has also resulted in a reduction in LA function [3–5] although the reports are conflicting, as several studies have demonstrated no change or even an improvement in LA function [6–8].

Among the three studies that have reported a reduction in LA function following catheter-based PV isolation, two were combined with linear lesions. One of these studies including linear lesions observed a reduction in LA ejection fraction (EF) as measured by ECG-gated computed tomography (CT) [3] while the other reported a reduction in LA EF as measured by magnetic resonance (MR) imaging already 48 days after the procedure which was limited to PV isolation and right atrial linear lesion [4]. The sensitivity of detecting a reduction in LA contractility may be higher with CT than with echocardiography and the additional lesion sets along the mitral isthmus and LA roof may have more extensive impact on LA function. Results are also difficult to interpret in small series of patients (*n* = 10) included [3] as compared with our study with 27 patients. Studies of atrial function made early after surgery may, however, reflect incomplete atrial remodeling, although the reduced atrial systolic function seemed strongly correlated with the volume of LA scar [4]. The third study reporting an impairment of LA function was an echocardiographic evaluation of left atrial emptying fraction mean 8 ± 2 months after a catheter-based PV isolation procedure in patients with paroxysmal AF [5].

Two other studies evaluating the same type of procedure in patients with paroxysmal AF [6] or paroxysmal and non-paroxysmal AF [8] reported no change in LA function as indicated by preserved LA active emptying fraction as measured by echocardiography [6] and by stable LA EF according to cardiac MR 12 months after PV isolation [8]. Others have reported an improved LA EF and a decrease in LA area (end systolic and end diastolic) in patients with symptomatic paroxysmal or persistent AF at 6 months after an AF ablation as assessed by cine electron beam computed tomography (EBCT) [7]. A significant decrease in LA size was also seen in patients with the largest LA at baseline in the present study and may either be related to the effect of remodeling after maintenance of sinus rhythm or as a result of the scar tissue shrinkage, the latter of which is contradicted by lack of effect on atrial contractility. Although, normal values for LA area [16] were recently presented, comparisons are not recommended as the literature is scarce [17]. We therefore preferred to refer to the standard LA diameter.

These diverse results observed in the literature may be related to the patient’s age, type of AF, the duration of follow-up, the type and the extent of ablation lesions, and also the methods used for evaluation. The previous studies have used diverse techniques for the evaluation of LA size and function, including echocardiography, EBCT, CT, or MR; comparisons of studies are difficult. Most of these imaging techniques are associated with limitations, for example, an asymmetric dilatation of the left atrium may under- or overestimate the LA area or volume. Echocardiographic calculations of LA volume rely on geometrical assumptions, which per se is an inherent limitation. CT may be more sensitive to detect small reductions in LA function but is associated with a burden of X-ray, and therefore not widely applied. A meta-analysis recently concluded that successful RF catheter ablation in patients with AF significantly decreases LA size and volumes but does not seem to adversely affect LA function [18]. Theoretically, the epicardial AF ablation approach might result in a more extensive scar since the RF clamp is applied well outside the PV pairs in the LA antrum. There are some studies examining the LA function after epicardial PV isolation and limited LA surgical ablation concomitant to cardiac surgery, a procedure that is difficult to compare with the results in our study since the cardiac surgery itself most likely affect the LA function [19–21].

Compier et al. [19] concluded that even limited LA ablation decreased LA volume, contraction, transport function, and compliance, indicating both reverse remodeling combined with significant functional deterioration. In contrast, surgical PV isolation alone decreased LA volume while function remained unchanged. They made limited LA ablation as a concomitant procedure for patients scheduled for valve surgery and/or coronary revascularization and had a control group consisting of patients undergoing concomitant epicardial PV isolation only. Signs of atrial dysfunction were also reported after combined mitral valve surgery and left atrial cryoablation for AF as opposed to with mitral valve surgery alone [21], although in that procedure extra linear lesions were also included.

Others, Buber J et al. [20], concluded that absence of LA contraction resulted in a fivefold increase and a LA volume index ≥ 33 ml/m^2^ in a threefold increase in the risk for thromboembolic stroke after the concomitant RF and cryoablation maze procedure, even when accounting for CHADS-VASc, in patients in sinus rhythm at 2 years follow-up.

According to our study, the mEPVI-GPabl does not seem to affect LA mechanical function or LA size, as assessed 6 months after the procedure. Neither were there signs of LA function deterioration with the extent of surgical ablation applications. The relatively small number of patients and the missing data may have affected the result.

As described previously [9], 76% had no symptomatic AF recurrences or AF episodes on 24-h Holter recordings at 12 months follow-up. The success rate is important since AF may cause significant structural remodeling and decreased contractility of the left atrium [22] promoting thromboembolism [23]. In our study, there were no thromboembolic events during 12 months follow-up except for in the one patient in whom an adequate anticoagulation medication had not been followed which emphasizes the importance of adequate anticoagulation throughout the procedure.

## Conclusion

In conclusion, the minimal invasive epicardial off-pump PV isolation combined with GP ablation resulted in no significant alteration of the atrial contractility, mechanical function, or size of the left atrium. Hence, this minimally invasive procedure with the elimination of AF recurrences and preserved atrial function may decrease the risk for thromboembolic events which may have implications for the risk evaluation of thromboembolic complications after this surgery.

## Limitations

Since there are multiple factors, including left atrial pressure, volume status, heart rate, and many others, which affect the E-wave, A-wave velocities, and E/A ratios as surrogates for mechanical function, it is difficult to draw conclusions regarding mechanical function based on these variables.
